# The intention of utilization and experience toward traditional Chinese medicine among breast cancer patients in the early and late stages: a qualitative study

**DOI:** 10.1186/s12906-023-04054-0

**Published:** 2023-07-07

**Authors:** Kai-wei Chen, Kuo-piao Chung, Chung-hua Hsu

**Affiliations:** 1 Institute of Health Policy and Management, National Taiwan University, 17 Xuzhou Road, Zhongzheng District, Taipei City, 100 Taiwan; 2 Linsen, Chinese Medicine and Kunming Branch, Taipei City Hospital, 100 Kunming Street, Wanhua District, Taipei City, 108 Taiwan

**Keywords:** Cancer, Traditional Chinese medicine, Stages, The intention of utilization and experience, Breast cancer

## Abstract

**Background:**

In Taiwan, breast cancer patients usually take conventional medicine and traditional Chinese medicine simultaneously. The utilization of traditional Chinese medicine among breast cancer patients at various stages has not been examined. This study aims to compare the intention of utilization and experience toward traditional Chinese medicine among early- and late-stage breast cancer patients.

**Method:**

This qualitative research collected data from breast cancer patients through focus groups interview by convenience sampling. Conducted in 2 branches of Taipei City Hospital, a public hospital managed by the Taipei City government. Breast cancer patients > 20 years old and had used TCM for breast cancer therapy for at least 3 months were included in the interview. A semi-structured interview guide was adopted in each focus group interview. In the following data analysis, stages I and II were considered early-stage, and stages III and IV were late-stage. For analyzing the data and reporting the results, we used qualitative content analysis as the approach for data analysis, assisted by NVivo 12. Categories and subcategories were identified through content analysis.

**Results:**

Twelve and seven early- and late-stage breast cancer patients were included in this study, respectively. The side effects were the main intention of utilizing traditional Chinese medicine. Improving side effects and constitution was the main benefit for patients in both stages. Additionally, early-stage breast cancer patients used traditional Chinese medicine to prevent recurrence or metastasis. Late-stage breast cancer patients responded more frequently to the use of traditional Chinese medicine due to the side effects of western medicine. However, some of their symptoms were not fully relieved.

**Conclusions:**

Breast cancer staging may influence the intention and utilization of traditional Chinese medicine. Health policymakers should consider the results of this research and the evidence-based illustrations to establish guidelines for integrating traditional Chinese medicine among various stages of breast cancer to improve the outcome and quality of care for cancer patients.

**Supplementary Information:**

The online version contains supplementary material available at 10.1186/s12906-023-04054-0.

## Introduction

Cancer is the leading cause of death worldwide, responsible for nearly 10 million deaths in 2020 [1]. In Taiwan, cancer has been the main cause of death for over 40 years [2]. In 2019, the incidence rate of colorectal and breast cancer were the highest among males and females, respectively, in Taiwan [3]. While breast cancer is the second most common cancer among all genders and the most common cancer among women worldwide [4, 5]. The age-standardized incidence rate of breast cancer in more developed regions is higher than in less developed regions [5, 6]. Besides, breast cancer was the second cause of death in developed countries in the world [4, 5]. In 2021, breast cancer is the fourth leading cause of cancer death, with a crude death rate of 24.6 per 100,000 population [7]. Due to the poor quality of life, anxiety, chronic pain, and psychosocial stress, cancer patients might use complementary and alternative medicine (CAM), to improve the symptoms after cancer diagnosis [8]. The prevalence of CAM use in cancer patients worldwide is approximately 40% [9], and the complementary therapies of cancer include Traditional Chinese medicine (TCM), diet supplements, herbal medicines, homeopathy, body-mind therapies, and others [10, 11]. TCM, which include herbal medicine, acupuncture, Qi Gong, Tai Qi, food therapy, Tui Na (Chinese massage) [12, 13], is the type of CAM mostly used in Taiwan [14]. According to the National Center for Complementary and Integrative Health(NCCIH), complementary medicine approaches can be classified as nutritional, psychological, physical, and combinations [15]. And due to TCM cannot fit any of these, it is defined as an “other complementary health approach” [15]. Despite it, TCM is still widely used because of the ancient Chinese culture and because it is an important medical approach in Taiwan [16]. All Taiwanese citizens are mandated to join the National Health Insurance, a single-payer system for healthcare implemented in 1995 [16, 17]. Its coverage rate in recent years is more than 99% [17, 18]. Under the National Health Insurance, Western medicine (WM) and TCM were included in the program [16, 17, 19]. People can choose WM, TCM, or both because of the accessibility and affordability of the healthcare system [16–19]. Conventional medicine, a system that involves medical doctors and other healthcare professionals to treat symptoms and diseases [20], is usually combined with TCM among cancer patients in Taiwan [21]. Studies have shown that TCM is most commonly used by patients with breast cancer (BCPs) [11]; 81.5% of BCPs will use TCM at least once after diagnosis, and 95.8% of TCM users used WM simultaneously [22]. The most frequently used TCM approaches for BCPs in Taiwan are Chinese herbal medicine (80.5%), followed by acupuncture or traumatology manipulative therapies (22.3%) [23]. However, according to research about the mental health of cancer patients, early- and late-stage patients have different degrees of depressive symptoms[24]. Patients with more advanced cancer stages were more likely to use Chinese medicine after diagnosis [25]. Besides, the effects of TCM care for cancer patients at different stages, including prognosis and survival time, vary [13, 26–28]. Therefore, because of the various stages of cancer, the intention of TCM utilization and experience may differ. There is a lack of studies exploring the intention of utilization and experience toward TCM among early- and late-stage cancer patients. Breast cancer has a high incidence rate, and BCPs tend to use TCM [3, 7, 11, 23]. This study compares the intention of TCM utilization and the experience of early- and late-stage BCPs.

## Method

### Design

To explore the intention and experience of early- and late-stage BCPs toward TCM, we adopted a qualitative research method. We collected and analyzed interview data regarding the participants’ perspectives from March to June 2021 by audio recording and analyzed the data after transcribing them verbatim. This study conducted focus group interviews for BCPs to understand the comparison between early- and late-stage breast cancer. In this study, stages I and II were considered early-stage, and stages III and IV were late-stage, based on the definition of the National Breast Cancer Foundation [29] and several studies [30, 31].

### Participants and setting

Nineteen BCPs participated in this study. In the focus group interviews, there were 3 to 5 participants in five focus groups, regarded as mini focus groups. With the interviewer’s guidance, the participants can provide more in-depth perspectives in a mini focus group [32]. Patients > 20 years old, diagnosed with breast cancer, and had used TCM for breast cancer therapy for at least 3 months were included in the interview. Before the interview, the interviewer informed the participants of their rights and that they could withdraw from the study anytime for any reason. The code numbers were applied instead of their names to maintain anonymity. The participants who agreed to the study signed an informed consent form. This study was approved by the Research Ethics Committee of Taipei City Hospital (Protocol number TCHIRB-11002011-E).

### Data collection

The participants were recruited using electronic and paper posters that were approved by the Research Ethics Committee of Taipei City Hospital. The posters were distributed among cancer patient groups at the hospital, with convenience sampling employed to collect the data. Following the recruitment process, BCPs were interviewed in several focus groups. We used a semi-structured guide with open-ended questions to conduct the interviews. The interview guide included six open-ended questions developed from the literature [33–35] and revised after discussing with co-authors (See the Additional file 1). The interviews were conducted in the Ren-ai Branch and Linsen Chinese Medicine and Kunming Branch of Taipei City Hospital, a public hospital with 4,700 beds in seven branches managed by the Taipei city government [36]. The data collection process continued until saturation was reached. The interviews were recorded using an audio recorder and subsequently transcribed into traditional Chinese text.

### Data analysis

After the interviews, the meaning unit of this study was found by listening to the audio record repeatedly and browsing the transcript verbatim followed by coding in accordance with the study’s objective after carefully understanding the text [37]. The data were analyzed through a conventional content analysis approach, which included driving the original codes after repeatedly reading the text and inducting or deducting them into subcategories, categories, and themes [38–40]. We applied computer-assisted qualitative data analysis software—Nvivo 12— to assist the organization and classification of data analysis and improve its efficiency [41]. To elevate the rigor of the qualitative study, triangulation, and peer review were conducted in the research. We also collected adequate data and increased the credibility, dependability, confirmability, and transferability through the thick description and maximum variation [41–43].

## Results

### Participants’ characteristics

Twenty-one BCPs were recruited for focus group interviews, but two were absent. Therefore, 12 and 7 BCPs in the early and late stages completed the interviews. The age of the participants ranged from 43 to 71 (mean = 55.42 ± 7.11) years. The mean time since cancer diagnosis was 3.92 ± 2.53 years (Table 1). In the following text, the codes starting with ‘E’ or ‘A’ refer to BCPs with early or late stages, respectively.

**Table 1:** Statistics on the information of breast cancer patients participants

Characteristic	No. (SD)
**Mean age**	55.42 (± 7.11)
**Time since diagnosed (year)**	3.92 (± 2.53)
**Stage**	
**Early stage**	
I	7
II	5
**Late stage**	
III	1
IV	6
**The class of hospital for TCM consulted**	
Regional	2
District	17

### The intention of TCM utilization for BCPs

BCPs were influenced by their feelings about diagnosis and multiple reasons for attempting TCM. According to data analysis, this theme contains two categories, including the mental state of BCPs after cancer diagnosis and the factors influencing BCPs to use TCM (Table 2).

**Table 2:** Key codes of the intention of TCM utilization for BCPs

Categories	Subcategories	Codes	Frequency of early-stage BCPs^a^	Frequency of late-stage BCPs^a^
Mental feelings of BCPs after cancer diagnosis	Negative mental impact	Cannot accept the diagnosis	8	4
		Shocked	9	6
		Sad or tearful	6	3
		Scared or upset	3	3
		Thought she was healthy	3	1
		Thought about the follow-up arrangements	1	3
	Facing it positively	Did not be nervous or worried	7	3
		Did not think it was an illness	2	0
		Soul had sustenance	1	1
		Peaceful coexistence	2	1
		Accepted it and faced it	3	4
		Hoped to live on	1	4
The influence factors of BCPs starting to use TCM	Push force of using TCM	Suffering from side effects	4	10
		Worried about the treatment of WM	1	1
	Pull force of using TCM	Recommendations from family members or friends	11	7
		Hoped to adjust her constitution	7	7
		Trust in TCM	2	2
		Based on personal experience	2	1
		Prevention of recurrence or metastasis	4	0
		WM recommendation	1	0
		Last resort	1	0

^a^Among the BCPs participants, there are 12 in the early stage and 7 in the late stage

#### Mental state of BCPs after cancer diagnosis

BCPs were shocked after diagnosis. Whether early or late-stage, most BCPs faced negative mental stress after a cancer diagnosis. Some BCPs could not accept the diagnosis and might be depressed, shocked, or upset.

*“At that time, it was really very upsetting, I couldn’t accept it, why did I get cancer?”* (E1)

Some BCPs faced the diagnosis head-on without nervousness or fear. They accepted it and lived peacefully because they believe cancer is not an illness. They also believe their soul has sustenance and hope to continue surviving.

*“My thought was that I could persist in (survive) or something like that, I can persist in till the end.”* (A1)

#### Factors influencing BCPs to use TCM

BCPs started using TCMs for various reasons. Under conventional treatment, BCPs usually suffer physical and psychological side effects, including skin symptoms, hot flashes, constipation, depression, insomnia, and restlessness. Some were also worried about the treatment with WM. While nearly all BCPs were plagued by conventional medical side effects that drove the force of pushing to TCM utilizing. Especially mentioned more frequently in late-stage patients.

*“Because of the side effects of the drugs at that time, I would have itching and hives on my body.”* (E11)

*“I used that TCM for conditioning, and then I adjusted my mentality, … Because of the side effects of chemotherapy, there would be hot flushes and insomnia… that was emotional anxiety.”* (A1)

However, BCPs used TCM might also be affected by the force of pulling. In other words, BCPs in any stage used TCM based on the recommendations of family members or friends, individual trust, and personal experience, and believed TCM could help to adjust the patients’ constitution.

*“At the beginning of chemotherapy, many friends introduced me to visit the TCM physician and suggested I see him.”* (A6)

*“I am quite confident in Chinese medicine. …, I was diagnosed in May 2010, …, I have been taking traditional Chinese medicine for conditioning since then.”* (E2)

Furthermore, early-stage BCPs believed that tumor recurrence or metastasis could be prevented through TCM utilization.

*“I had been worrying about whether the tumor will recur, metastasize, or I will die,… then I went to see the TCM physicians.”* (E1)

### The experience of BCPs in TCM care

Under TCM care, BCPs experienced some changes. According to data analysis, this comprises two categories: the perceived effects and feelings of TCM and the expectations for TCM care (Table 3).

**Table 3:** Key codes of the experience of BCPs in TCM care

Categories	Subcategories	Codes	Frequency of early-stage BCPs^a^	Frequency of late-stage BCPs^a^
Perceive effects and feelings of BCPs from TCM experience	Changes in physiology	Relieving side effects	15	10
		Constitution enhancement	12	3
		Tumor Control and Prevention	4	1
		Improved physiological function	6	2
		Improved the quality of life	1	0
		Reduced the dose of WM	1	0
		Catastrophic illness has been canceled	2	0
	Changes in psychological	Felt peace of mind	9	3
		Mood was better	1	0
	Changes to social well-being	Improved family relationships	1	0
	Unmet care needs	Side effects were not fully been improve	1	3
	Communica-tion between TCM and WM	WM agreed with the use of TCM	4	2
		WM rejected TCM use	5	3
		Did not inform WM physicians	3	3
The expectation of BCPs to TCM care	Clinical practices of TCM	Medication Safety and Convenience	5	2
		Establish TCM consultation	5	0
		Long waiting time	3	4
	Policies of TCM	Suggestions for scientific TCM	2	0
		Cooperation between Chinese and Western medicine	1	0
		Suggestions for daycare of cancer	3	8

^a^Among the BCPs participants, there are 12 in the early stage and 7 in the late stage

#### Perceived effects and feelings of TCM

Early- and late-stage BCPs experienced the physical or psychological perceived effects of TCM.

Early- and late-stage BCPs frequently mentioned that TCM improved the side effects of their conventional medical treatment. They also mentioned the enhancements to their body constitution.

*“The biggest improvement for me from TCM is that I used to have diarrhea, … but after taking this Chinese medicine, it became normal.”* (E12)

*“In addition to fatigue, it was rash, diarrhea, and hand-foot syndrome. I think the acupuncture from daycare of TCM is very helpful for me.”* (A2)

Furthermore, after TCM care, BCPs felt more spiritually stable and had improved moods. Early- and late-stage BCPs had similar perspectives.

*“After taking the Chinese medicine, it let me sleep better, and then I felt calmer.”* (A1)

*“I feel that Chinese medicine decoction gives me a very stable strength. …. I believe that if my mental status is healed, then my body should also be fine.”* (E4)

However, some BCPs mentioned that their needs for using TCM were not being met. Especially late-stage BCPs frequently mentioned that their side effects were not relieved.

*“I think insomnia is actually very difficult to relieve …every time I told the physician: ‘I can’t sleep’, he would change the medicine and strengthen it.*” (A7)

For the use of TCM, regardless of early- or late-stage breast cancer, patients communicated with WM practitioners and either got approval or were rejected. Moreover, some BCPs did not inform WM physicians of TCM usage due to fear or feeling that it was meaningless.

*“The western physician said, ‘okay, if it helps you, just go to see TCM.”* (E11)

*“I already trust TCM very much, I don’t need WM to give me affirmation, so I don’t need to ask them.”* (E2)

#### The expectations for TCM care

Most BCPs were satisfied with the current TCM care but had some expectations about TCM. In clinical practice, many BCPs mentioned that the safety and convenience of TCM still need to be strictly verified. The long waiting time and the lack of available TCM consultations should be improved.

*“The ingredients of concentrated scientific Chinese medicine is the toxicity of lead. Many people will worry about this problem.”* (A1)

*“There are really too many patients in the TCM physician…the time to answer my question may be a bit rushed.”* (A6)

In terms of policy, BCPs thought TCM should be more scientific and incorporated with WM. The enhanced daycare of TCM for cancer can be implemented more extensively.

*“I feel that Chinese medicine is going to develop this kind of instrument that can be digitized…that can help them make a more accurate diagnosis.”* (E9)

*“I think this enhanced daycare of TCM is only available in our hospital. I think it is too lack, and it really needs to be everywhere.”* (E10)

## Discussion

To our knowledge, this is the first study to explore the intention and experience of BCPs in the early and late stages toward TCM utilization using content analysis to compare the frequency of specific codes. Through this study, we can further understand the factors that affect BCPs’ demand for TCM care.

### BCPs were driven by push and pull by cancer diagnosis and treatment to use TCM

The intention of BCPs to use TCM in this study may be a disturbance to the disease, which becomes a push for the use of TCM; BCPs believed that TCM could benefit cancer referred to as a pull for using TCM.

Previous studies have indicated that patients will generate push factors for TCM use due to dissatisfaction with WM care or generate pull factors based on their background and attraction to the benefits of TCM care, forming a “push-pull model” [34, 44].

This study found that suffering from the side effects of WM treatment is an important push force driving early- and late-stage BCPs to use TCM. Late-stage BCPs especially mentioned this disturbance more frequently and hoped TCM would strengthen their constitution. It is similar to the results of previous studies [22, 45, 46] in that BCPs used TCM to improve the side effects, provide tonic effects, and treat the tumor since late-stage BCPs might experience more side effects after WM treatment, including physical and psychological discomfort. Hamer et al. demonstrated that advanced BCPs had greater symptom burden and lower quality of life than early-stage BCPs [47]. Therefore, late-stage BCPs had more motivation to undergo TCM. In addition, early- and late-stage BCPs use TCM because of the trust and hope that TCM can adjust their body constitution, which is common between both groups. Hence, cancer patients usually trust TCM because of their cultural background and personal experience [35].

However, the factor “hoping to prevent metastasis and recurrence” was only mentioned by early-stage BCPs. It may relate to the results of this study. Late-stage BCPs do not aim to cure or eliminate cancer but hope that TCM can improve their physical strength and reduce side effects so they can coexist peacefully with cancer cells. One literature pointed out that early-stage BCPs experience crucial psychological distress regarding recurrence compared with late-stage BCPs [48].

An early-stage BCP responded that she thought TCM was the last option except for WM, so she tried to receive TCM care. TCM can be regarded as a BCP’s last resort to stay alive, according to Smithson et al. and Yu et al., seeking CAM or TCM is the last resort for cancer patients to control cancer and get better outcomes [33, 49].

### TCM is beneficial for breast cancer care, and BCPs in various stages had different care needs for TCM

The most frequent response was that TCM could improve the side effects of the BCPs, whether early- or late-stage, and enhance physical strength so they can continue WM treatment. Most participants only use oral Chinese medicine, including concentrated powder and Chinese medicine decoction. Some participants also receive acupuncture treatment in addition to obtain more intensive TCM care. In participants’ responses, TCM can improve physiological side effects such as diarrhea or constipation, insomnia, skin rash, poor appetite, hot flashes, menopausal syndrome, hand-foot syndrome, and symptoms of numbness in the hands and feet. Especially, participants in enhanced daycare indicated significant improvements not only in relieving gastrointestinal symptoms induced by WM but also in reducing fatigue and enhancing physical strength after TCM intervention. The improvement in quality of life was also significant and believed to be beneficial. On the other hand, TCM can also play a role in enhancing psychological well-being. Almost all participants mentioned feeling calm and experiencing an improved mood after receiving TCM intervention. Many studies have demonstrated that TCM can alleviate the adverse effects of conventional medicine [12, 45, 50–53], inhibit cancer cells [54, 55], and improve the quality of life or prolong survival time [13, 51, 54–56].

According to the participants’ responses, TCM has different effects in different periods of WM treatment. Before WM treatment, TCM plays a preventive role. During the WM treatment period, both early- and late-stage BCPs are suffered by the side effects of WM, and TCM can relieve their symptoms and help the treatment continue and complete. After finishing the WM treatment, TCM becomes a supportive and maintenance role, allowing patients to return to their normal lives.

**Fig. 1:**
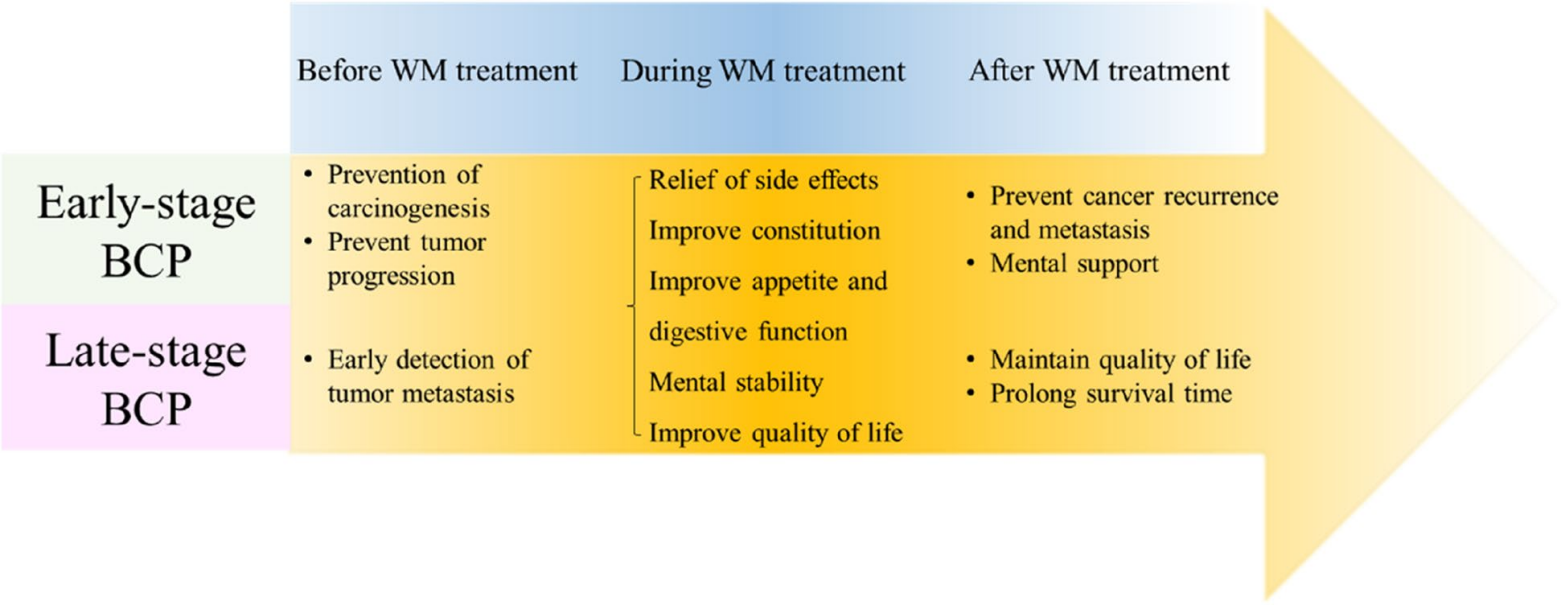
TCM care has different effects on early- and late-stage BCPs during various WM treatment periods

‘Tumor control and prevention’ is one of the positive changes brought about by TCM care, and early-stage patients mentioned it frequently. Compared with late-stage breast cancer, early-stage breast cancer cells are more stable and can be protected from tumor progression through TCM. Some previous studies have reported that one of the reasons why cancer patients use TCM is to prevent tumor recurrence or metastasis [34]. TCM has proven to be effective in preventing cancer recurrence and metastasis [55, 57]. A study showed that the rates of psychosocial distress are high and similar among early- and late-stage BCPs [58], and TCM can improve psychological stability in both groups. The effects on early- and late-stage BCPs during various WM treatment periods had shown in Fig. 1. In particular, one participant mentioned that after TCM care, she could take care of herself, and her family relationship improved. This is like the World Health Organization’s definition of health: ‘health is a state of complete physical, mental, and social well-being and not merely the absence of disease or infirmity’ [59]. However, after TCM care, some participants felt that their care needs regarding side effects had not been met, and most were late-stage patients. It is estimated that the treatment methods and doses for late-stage BCPs are more complicated when treated with WM, making the side effects more pronounced, so the need for TCM would also be affected. This response is similar to a previous study [60] indicating that cancer patients in late stages generally undergo chemotherapy and radiotherapy as the preferred treatments after surgery. While the side effects of these therapies, including myelosuppression, gastrointestinal tract reactions, and cardiac damage, can be more complicated. Moreover, tumor cells may develop resistance to conventional treatments, rendering them less effective over time [60]. Therefore, TCM can serve as an adjunctive therapy, making efforts to deal with the side effects and complications caused by chemotherapy and radiotherapy for BCPs in late stages.

TCM is beneficial to BCPs, and some WM practitioners agreed for the use of TCM, especially for early-stage breast cancer. However, many BCPs responded that WM practitioners were less accepting of TCM due to worries about the mutual influence of Chinese and Western medicine. WM consultants of late-stage BCPs were more reluctant to accept TCM, probably because they were more worried about its possible interaction with efficacy. Not all BCPs discussed the use of TCM with WM, probably because of fear of telling or the feeling that reporting it is meaningless. Studies have also shown that many BCPs do not inform WM physicians of the use of CAM or TCM, and most of their WM physicians were unaware that the patients were using other therapies [61, 62]. Therefore, the risk of potential interaction between Chinese and Western medicines may be increased, causing unexpected adverse reactions [22, 63].

Some participants believe that WM and TCM should be integrated to establish communication and cooperation. Many studies have shown that proper integration of Chinese and Western medicine in cancer care can improve the survival rate, quality of life, and overall health of cancer patients [13, 22, 64–66]. Wang and his colleagues [65] conducted a meta-analysis study on 29 randomized controlled trials studies that included 3,142 BCPs. The study found that compared to conventional treatments alone, the combination of TCM and conventional treatments can improve short-term treatment efficacy and have a better 3-year and 5-year survival rate after mastectomy. It can also reduce the incidence of adverse reactions, including nausea and vomiting, leucopenia, thrombocytopenia, and upper extremity edema. Another meta-analysis study that included 22 randomized controlled trial studies with a total of 1,689 BCPs also confirmed that the combination of TCM interventions such as Chinese herbal medicine and acupuncture with conventional treatments can improve the quality of life, alleviate side effect symptoms, and improve the value of tumor markers, compared to those who use conventional chemotherapy, radiation therapy, and endocrine therapy alone [66]. In this study, participants believed that if TCM clinics had more time for consultations, TCM could be practiced in a more scientific manner and the quality of TCM treatments could be improved, resulting in safer care and better quality of life for cancer patients. Regarding the waiting time that some BCPs expect, due to the coverage of TCM by the National Health Insurance in Taiwan, Approximately 5.5 million individuals sought TCM outpatient care in 2021, resulting in an average of 6.24 visits per user [67, 68]. Notably, cancer patients specifically chose TCM care based on their reputation [35]. Consequently, this heightened demand can potentially lead to prolonged waiting periods at certain TCM clinics. As stated in the literature, scientific evidence-based research on TCM should be strengthened [46, 53, 56], and the heterogeneity of Chinese medicine quality should improve [53], since the enhanced daycare of TCM for cancer is also beneficial to the alleviation of adverse reactions for cancer patients [69].

### Limitations

The BCPs focus group interviews in this study included 19 participants, 12 early-stage and 7 late-stage patients. Although great efforts were made for recruitment, the numbers of early- and late-stage BCPs were unequal. The late-stage BCPs were fewer, similar to the breast cancer survival statistics, and this study tried to encourage participants to fully express their opinions during the interviews. This study recruited BCP participants by convenience sampling, which may introduce a selection bias. While the viewpoints were completely collected in the research until it was saturated, future research should extend the sampling source to avoid possible bias.

According to the study’s purpose, we only included those who had experience using TCM; we did not explore the views of non-users. Therefore, the reasons why those BCPs did not use TCM care are unknown. We only explored participants’ utilization and experiences of TCM in the healthcare system and did not discuss the types and frequency of TCM used by participants. Future research may be needed to further explore the above.

## Conclusion

Relieving the side effects of conventional treatment and trust in TCM are the main factors for BCPs to use TCM. Many BCPs experienced relief from side effects, improved constitution, and emotional stability with TCM. Early-stage BCPs additionally expected that TCMs could prevent cancer from recurrence and metastasis. Late-stage BCPs frequently indicated that the need for care was not fulfilled. Early- and late-stage BCPs’ intentions and experiences in TCM utilization were inconsistent. Scientific evidence-based research on TCM and integration with WM to improve the quality of cancer care should be strengthened. Based on the results of the studies, health policymakers can appropriately revise cancer TCM care programs or care guides with WM to meet the treatment needs of various stages to improve the quality of care and outcome in cancer care.

## Supplementary Information

**Additional file 1.** Interview guide for BCPs focus groups.

## Data Availability

The datasets generated and analyzed during the current study are not publicly available due to the informed consent which stated that the relevant data in this research was available for the authors in this study only but are available from the corresponding author on reasonable request.

## Abbreviations

CAM: Complementary and alternative medicine
TCM: Traditional Chinese medicine
BCPs: Patients with breast cancer
WM: Western medicine
