# Supplementary material for: The intention of utilization and experience toward traditional Chinese medicine among breast cancer patients in the early and late stages: a qualitative study
Source: BMC Complement Med Ther. 2023 Jul 7;23:226. doi: 10.1186/s12906-023-04054-0 (PMC10329334; doi:10.1186/s12906-023-04054-0)
Supplement: Supplementary file 1 — Additional file 1. Interview guide for BCPs focus groups. [file 12906_2023_4054_MOESM1_ESM.docx]

# Additional file 1: Interview guide for BCP focus groups

|  | Semi-structured interview guides |
| --- | --- |
| 1 | Please introduce yourself briefly. |
| 2 | How did you feel when you were diagnosed with cancer and its stage? |
| 3 | Under what situation would you start using TCM after the diagnosis? |
| 4 | What do you think is the effect of using TCM on cancer? |
| 5 | If you also visit WM, how will you discuss the use of TCM with the Western doctor? |
| 6 | In your current experience of using TCM, if you think that there are deficiencies in healthcare resources or something for the suggestion, please provide your opinions. |
